# PINK1 and Parkin cooperatively protect neurons against constitutively active TRP channel-induced retinal degeneration in *Drosophila*

**DOI:** 10.1038/cddis.2016.82

**Published:** 2016-04-07

**Authors:** Z Huang, S Ren, Y Jiang, T Wang

**Affiliations:** 1 State Key Laboratory of Molecular Developmental Biology, Institute of Genetics and Developmental Biology, Chinese Academy of Sciences, Beijing 100101, China; 2 National Institute of Biological Sciences, Beijing, China; 3 University of Chinese Academy of Sciences, Beijing 100049, China; 4 College of Biological Sciences, China Agricultural University, Beijing, China

## Abstract

Calcium has an important role in regulating numerous cellular activities. However, extremely high levels of intracellular calcium can lead to neurotoxicity, a process commonly associated with degenerative diseases. Despite the clear role of calcium cytotoxicity in mediating neuronal cell death in this context, the pathological mechanisms remain controversial. We used a well-established *Drosophila* model of retinal degeneration, which involves the constitutively active TRP^P365^ channels, to study calcium-induced neurotoxicity. We found that the disruption of mitochondrial function was associated with the degenerative process. Further, increasing autophagy flux prevented cell death in *Trp*^*P365*^ mutant flies, and this depended on the PINK1/Parkin pathway. In addition, the retinal degeneration process was also suppressed by the coexpression of PINK1 and Parkin. Our results provide genetic evidence that mitochondrial dysfunction has a key role in the pathology of cellular calcium neurotoxicity. In addition, the results demonstrated that maintaining mitochondrial homeostasis via PINK1/Parkin-dependent mitochondrial quality control can potentially alleviate cell death in a wide range of neurodegenerative diseases.

As a key messenger, cellular calcium regulates multiple neuronal functions, while the dysregulation of calcium homeostasis ultimately leads to neurotoxicity.^1, 2, 3^ Both clinical and experimental evidence suggests that calcium-activated cell death has a central role in the pathogenesis of a variety of neurological diseases, including stroke, Alzheimer's disease, Parkinson's disease, Huntington's disease, and amyotrophic lateral sclerosis.^4, 5^ Although it is well known that increased levels of cytoplasmic calcium can trigger a range of downstream neurotoxic cascades, the main pathological signaling events downstream of calcium overload remain unclear. In addition, pharmacological treatments to suppress calcium-induced cell death are still lacking.

Mitochondria are the major source of cellular energy, producing adenosine triphosphate (ATP) through oxidative phosphorylation. In the case of neurons, almost all ATP is generated by mitochondria.^6^ Dysfunctional mitochondria not only disrupt the supply of cellular ATP, but also release pro-apoptotic proteins and toxic reactive oxygen species (ROS), which ultimately lead to cell death.^7, 8^ Further, mitochondria contribute to the maintenance of cellular calcium homeostasis, while mitochondrial functions including ATP synthesis are regulated by calcium in the mitochondrial matrix.^9^ However, mitochondrial calcium overload, which is associated with mitochondrial fragmentation, mitochondrial membrane potential collapse, ATP depletion, accumulation of ROS, and the release of pro-apoptotic factors, eventually causes cell death.^2, 4, 10, 11, 12^ Therefore, cellular calcium overload ultimately impairs the ability of mitochondria to contribute to metabolism and energy production by disrupting calcium homeostasis.^9^

Eukaryotic cells have several mechanisms with which to maintain mitochondrial homeostasis and hence their function and survival.^13^ Damaged mitochondria are identified and eliminated through a mitochondrial quality control pathway known as mitophagy, which involves the selective autophagic removal of mitochondria.^14^ Two factors, including a mitochondrially targeted protein kinase and an E3 ubiquitin ligase (encoded by the early-onset Parkinson's disease genes *pink1* and *parkin*, respectively) are key mediators of this mitochondrial quality control pathway.^15, 16, 17^ Damaged mitochondria, which are characterized by losses in mitochondrial membrane potential, exhibit stabilized PINK1 on the mitochondrial outer membrane. This stabilized PINK1 recruits and activates Parkin, which in turn triggers autophagy and the lysosomal degradation of dysfunctional mitochondria.^18, 19, 20, 21^

The *Drosophila* visual system has served as a powerful model for investigating the mechanisms that underlie retinal degeneration, as well as a variety of neurodegenerative diseases.^22, 23, 24, 25^ Importantly, genetic models of calcium overload-induced neurodegeneration have been well established in this system.^26, 27^ Here, we found that abnormal mitochondrial morphology and function were associated with the *Trp*^*P365*^ mutant, which expresses a constitutively active version of the *Drosophila* calcium channel involved in phototransduction. These mutant channels induce cell death by elevating cellular calcium levels.^27^ The upregulation of autophagy suppressed the retinal degeneration phenotype associated with the *Trp*^*P365*^ mutation, and both PINK1 and Parkin were required for this cell-protective effect. Further analysis revealed that Parkin was recruited to the mitochondria of *Trp*^*P365*^ mutant photoreceptor cells, and that the coexpression of PINK1 and Parkin prevented Trp^P365^-induced photoreceptor cell degeneration. These results suggest that the death of *Trp*^*P365*^ mutant photoreceptor cells primarily resulted from the disruption of mitochondrial function caused by calcium overload. Further, the PINK1/Parkin-dependent mitochondrial quality control pathway alleviated the retinal degeneration associated with the *Trp*^*P365*^ mutation.

## Results

### Cells that degenerate as a result of elevated cellular calcium contain dysfunctional mitochondria

**Figure 1:**
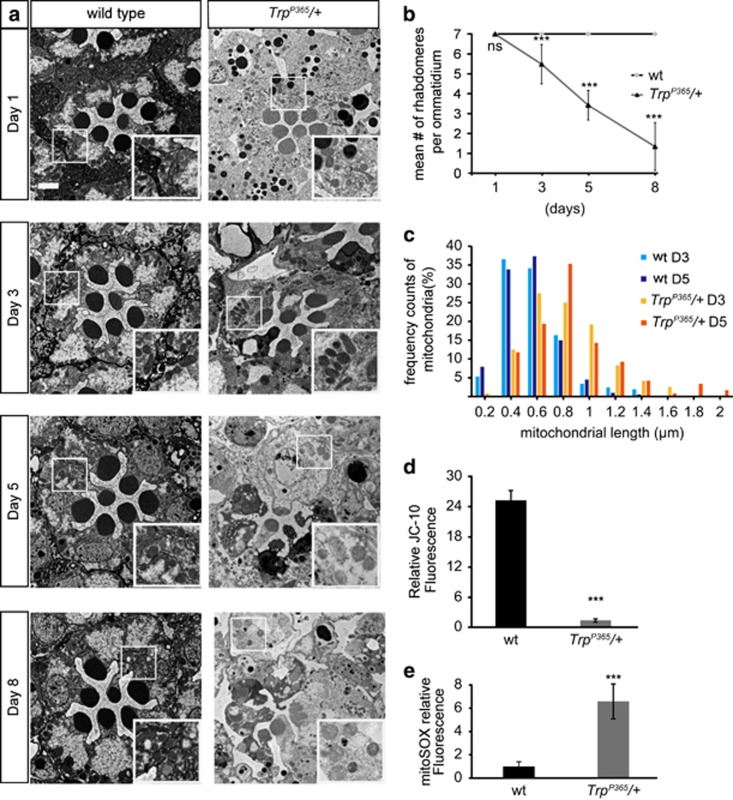
Retinal degeneration and mitochondrial dysfunction associated with *Trp*^*P365*^ mutant flies. (**a**) TEM images of single ommatidium from wild-type and *Trp*^*P365*^mutant flies (*Trp*^*P365*^/+) at indicated ages. High magnification views of the mitochondrial morphologies are shown in the boxed regions. Scale bar, 2 *μ*m. (**b**) Quantification of the mean number of rhabdomeres per ommatidium indicates the time course of retinal degeneration in *Trp*^*P365*^ flies. At least 150 ommatidia from three flies were counted for each point. (**c**) The distribution of mitochondrial length within photoreceptor cells. At least 200 photoreceptor cells of wild-type and *Trp*^*P365*^ flies at the ages of 3 and 5 days (D3 and D5) were evaluated to determine the mitochondrial length. (**d**) The retinal mitochondrial membrane potential was measured using JC-10 staining. The ratio of JC-10 fluorescence emission at 525 nm and 590 nm was calculated, and the relative membrane potential was normalized to the *Trp*^*P365*^ mutant. (**e**) Mitochondrial superoxide levels were measured via mitoSOX staining of dissected retina. At least five samples from 3-day-old flies were used. The mitoSOX fluorescence intensity was normalized to *Trp*^*P365*^ mutants. Significant differences were determined using Student's *t*-test at indicated age (ns, not significant; ***P<0.001)

The *Drosophila* gene *trp* (transient receptor potential) encodes a major calcium channel involved in phototransduction.^28^ The dominant *trp* allele, *Trp*^*P365*^, encodes a constitutively active TRP^P365^ channel, resulting in increased levels of cellular calcium and retinal degeneration.^26, 29^ This allele therefore provides a good model for investigating the mechanisms by which elevated cellular calcium causes cell death. In order to understand the initial cellular changes in *Trp*^*P365*^ mutant cells, we used transmission electron microscopy (TEM) to characterize the subcellular structures within photoreceptor cells of different ages. Consistent with the findings of previous work,^26^ *Trp*^*P365*^ heterozygous mutants exhibited a loss of photoreceptor cells during aging (Figure 1a). When viewed in tangential sections, the fly ommatidium includes seven photoreceptor cells. Each photoreceptor cell contains a central photoreception organelle, the rhabdomere (Figure 1a). Young *Trp*^*P365*^ flies exhibited normal eye morphology, whereas aged flies exhibited rapid degeneration with condensed cytoplasm, vesicle accumulation, and loss of rhabdomeres. In contrast, the wild-type controls exhibited normal eye morphology, with all seven rhabdomeres, regardless of age (Figures 1a and b).

In addition to the degeneration phenotype, the *Trp*^*P365*^ photoreceptor cells from both 3- and 5-day-old flies contained morphologically abnormal mitochondria. These mitochondria were larger and more vacuolated when compared with the controls, which was more severe at day 5 than day 3 (Figure 1c). To functionally characterize the mitochondria within these mutant cells, we assessed the mitochondrial membrane potential (i.e., the driving force of ATP synthesis) with JC-10 staining. The E595/E525 ratio of wild-type cells was ~25 times higher than that for *Trp*^*P365*^ photoreceptor cells in 3-day-old flies (Figure 1d). As impaired mitochondria generally produce increased levels of mitochondrial ROS, we measured the superoxide levels in *Trp*^*P365*^ mitochondria via mitoSOX. The relative mitoSOX fluorescence in the *Trp*^*P365*^ mutant cells was ~7 times higher than that in the wild-type cells, indicating that the mitochondria within the *Trp*^*P365*^ mutant cells are damaged (Figure 1e). These results suggest that the *Trp*^*P365*^ mutation affected mitochondrial function during the neurodegeneration process.

### Increased mitochondrial calcium uptake enhanced *Trp*^*P365*^-associated cell death

**Figure 2:**
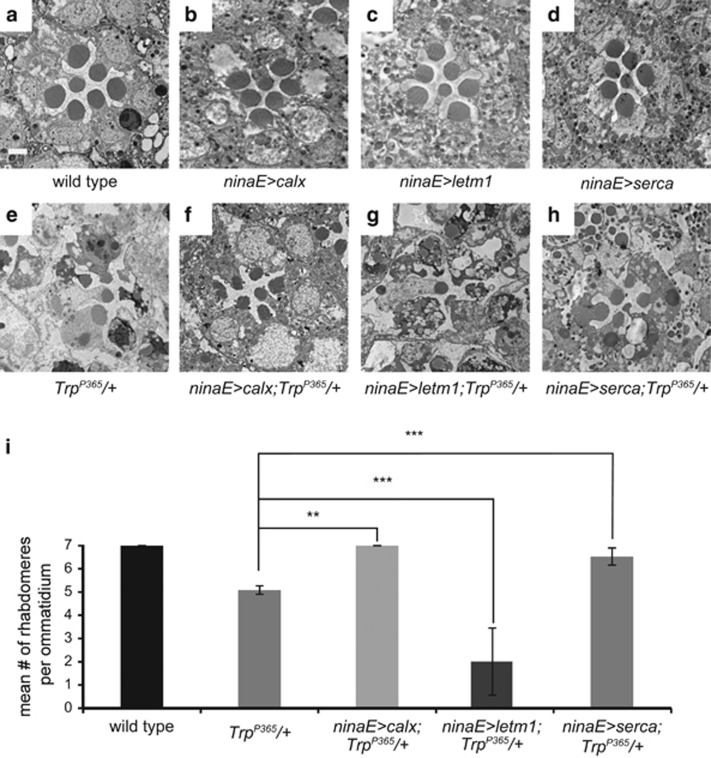
Letm1 enhances retinal degeneration in *Trp*^*P365*^ mutant flies. (**a–h**) Morphology of 5-day-old (**a**) wild-type, (**b**) *ninaE>calx*: *ninaE-gal4/UAS-calx*, (**c**) *ninaE>letm1*: *ninaE-gal4/UAS-letm1*, (**d**) *ninaE>serca*: *ninaE-gal4/UAS-serca*, (**e**) *Trp*^*P365*^/+, (**f**) *ninaE>calx*;*Trp*^*P365*^/+, (**g**) *ninaE>letm1*;*Trp*^*P365*^/+, and (**h**) *ninaE>serca*;*Trp*^*P365*^/+ flies. Scale bar, 2 *μ*m. (**i**) Mean number of rhabdomeres per ommatidium from 5-day-old flies. Quantifications were based on more than 150 ommatidia from three EM sections for each genotype. Error bars indicate S.E.M. (**P<0.01; ***P<0.001)

As previously reported, overexpression of the reciprocal Na^+^/Ca^2+^ exchanger CalX largely suppressed retinal degeneration in the *Trp*^*P365*^ mutant background, which suggests that the degeneration of *Trp*^*P365*^ mutant photoreceptor cells results from increased cellular calcium^26, 29^ (Figures 2a, b, e, and 2f). Mitochondria serve as large and dynamic physiological buffers for calcium, which is important in regulating mitochondrial function.^9^ We therefore reasoned that the increased level of cellular calcium associated with the constitutively active TRP^P365^ channels might disrupt mitochondrial function by dysregulating mitochondrial calcium. If the dysregulation of mitochondrial calcium contributes to retinal degeneration in *Trp*^*P365*^ mutant flies, then cell death may be enhanced by increased mitochondrial uptake of calcium. To test this hypothesis, we overexpressed LETM1 (leucine zipper-EF-hand containing transmembrane protein 1), the mitochondrial Ca^2+^/H^+^ antiporter responsible for mitochondrial calcium entrance, in photoreceptor cells using a very strong photoreceptor cell promotor, namely the *ninaE* promotor.^30^ Although the overexpression of LETM1 did not cause retinal cell death in wild-type flies, the death of *Trp*^*P365*^ photoreceptor cells was enhanced by the LETM1 overexpression (Figures 2c, g, and i). Moreover, upregulation of the calcium transport of endoplasmic reticulum (ER), the most important intracellular calcium store, by overexpression of an ER calcium pump SERCA also significantly suppressed in the *Trp*^*P365*^-associated degeneration (Figures 2d, h, and i). These interactions between a variety of calcium transporters and TRP^P365^ indicate that increased levels of mitochondrial calcium may have a key role in the pathology induced by high cellular calcium levels.

### TRP^P365^-induced cell death was suppressed by autophagy

**Figure 3:**
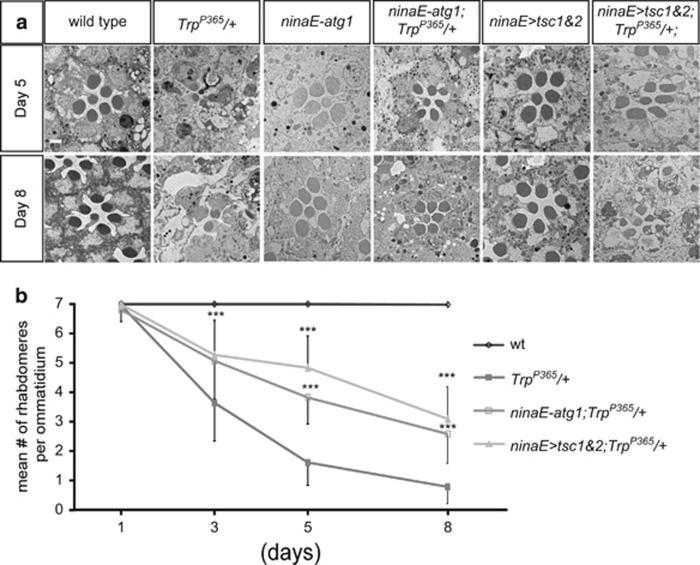
Activation of autophagy suppressed neural degeneration in the *Trp*^*P365*^ mutant flies. (**a**) TEM images of single ommatidium from wild-type, *Trp*^*P365*^/+, *ninaE-atg1*, *ninaE-atg1*;*Trp*^*P365*^*/+*, *ninaE>tsc1&2*(*ninaE-gal4/UAS-tsc1,UAS-tsc2*), and *ninaE>tsc1&2*; *Trp*^*P365*^/+ flies. Scale bar, 2 *μ*m. (**b**) Time course of photoreceptor degeneration. The number of rhabdomeres per ommatidium was examined by optical neutralization assay. Quantification was based on >150 ommatidia from three eyes from flies with the indicated genotypes and ages. Error bars indicate S.E.M. Significant differences between against *ninaE-atg1*;*Trp*^*P365*^*/+* or *ninaE>tsc1&2*;*Trp*^*P365*^/+ and *Trp*^*P365*^/+ were determined using Student's *t*-test under indicated age (**P<0.01; ***P<0.001)

Autophagy degrades intracellular aggregates, cellular debris, and damaged organelles, thereby protecting against a variety of cell death signals associated with neurodegenerative diseases.^31, 32^ We therefore tested whether the activation of autophagy could modify the severity of cell death in *Trp*^*P365*^ mutant flies. In order to induce autophagy, we overexpressed *atg1* via the *ninaE* promoter.^33, 34^ Compared with the *Trp*^*P365*^ flies, which lost most major photoreceptor neurons after 5 days, the *ninaE-atg1*/*Trp*^*P365*^ flies retained most of their photoreceptor cells when raised in identical conditions (Figures 3a and b). We then activated autophagy through the genetic inactivation of TORC1, the negative regulator of autophagy. TSC1 (tuberous sclerosis complex 1) and TSC2 (tuberous sclerosis complex 2) form a GTPase-activating protein for Rheb GTPase, thereby negatively regulating TORC1 kinase activity and activating autophagy.^34, 35, 36^ As seen with Atg1, the overexpression of TSC1 and TSC2 (*ninaE>Tsc1/2*) largely suppressed the neural degeneration caused by the *Trp*^*P365*^ mutation (Figures 3a and b). Therefore, the upregulation of autophagy prevented Trp^P365^-induced photoreceptor cell degeneration.

### PINK1 and Parkin are required for Atg1-mediated suppression of neurodegeneration in *TRP*^*P365*^ mutants

**Figure 4:**
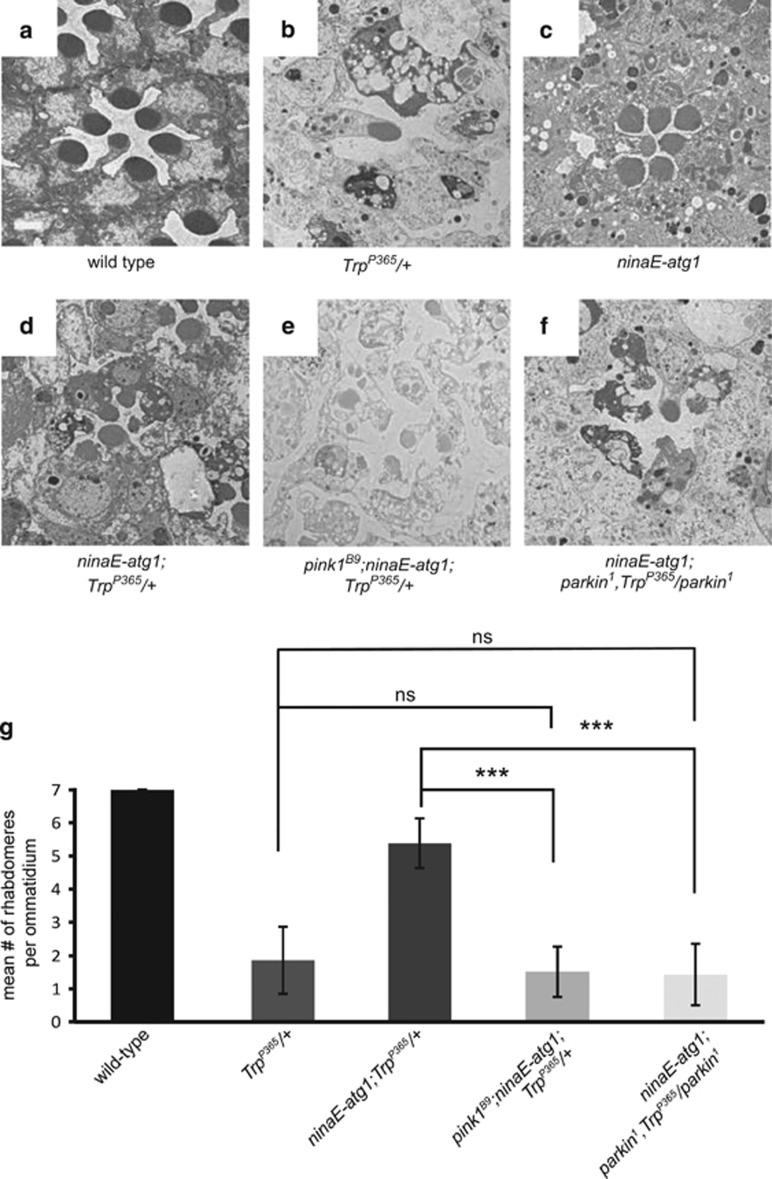
PINK1 and Parkin are required for Atg1 to suppress photoreceptor cell death in *Trp*^*P365*^ mutant flies. (**a**–**f**) Examination of single ommatidia by TEM. Cross-sections were obtained from 8-day-old (**a**) wild-type, (**b**) *Trp*^*P365*^/+, (**c**) *ninaE-atg1*, (**d**) *ninaE-atg1*;*Trp*^*P365*^/+, (**e**) *pink1*^*B9*^ *ninaE-atg1*;*Trp*^*P365*^/+, and (**f**) *ninaE-atg1*;*park*^*1*^*Trp*^*P365*^/*park*^*1*^ flies. Scale bar, 2 *μ*m. (**g**) The mean number of rhabdomeres per ommatidium was quantified from at least 150 ommatidia from three TEM sections for each genotype. Error bars indicate S.E.M. (ns, not significant; ***P<0.001)

The digestion of oxidatively damaged organelles by autophagy prevents the progressive accumulation of dysfunctional mitochondria and other organelles during aging. As *Trp*^*P365*^ mutant cells contain large numbers of defective mitochondria, it is reasonable to assume that mitochondrial autophagy is the primary mechanism by which elevated levels of autophagy suppress TRP^P365^-induced cell death. Indeed, overexpressing Atg1 restored mitochondrial morphology in *Trp*^*P365*^ retinal cells (Supplementary Figure S1). The mitochondrially targeted protein kinase PINK1 and the E3 ubiquitin ligase Parkin have central roles in mitophagy, which is the autophagic degradation of dysfunctional mitochondria.^20, 37^ Moreover, the induction of general autophagy by Atg1 enhances PINK1/Parkin-mediated mitophagy in both flies and mammals.^38^ Therefore, overexpression of Atg1 may prevent cell degeneration by increasing the autophagic removal of damaged mitochondria. To test this hypothesis, we overexpressed Atg1 in the *pink1*^*B9*^ and *Trp*^*P365*^ double mutant background. Strikingly, the loss-of-function mutation in *pink1* completely blocked the ability of Arg1 to protect the *Trp*^*P365*^ mutant cells (Figures 4a–e and g). We next introduced the loss-of-function *parkin* mutation, *park*^*1*^, into the *ninaE-atg1*/*Trp*^*P365*^ flies. As seen with the *pink1* loss of function, *park*^*1*^ also blocked the ability of Arg1 to protect the *Trp*^*P365*^ photoreceptor cells (Figures 4f and g). These results suggest that Atg1 prevents Trp^P365^-induced retinal degeneration by increasing the selective autophagy of mitochondria.

### PINK1 and Parkin cooperatively prevent cell death in *Trp*^*P365*^ mutant cells

**Figure 5:**
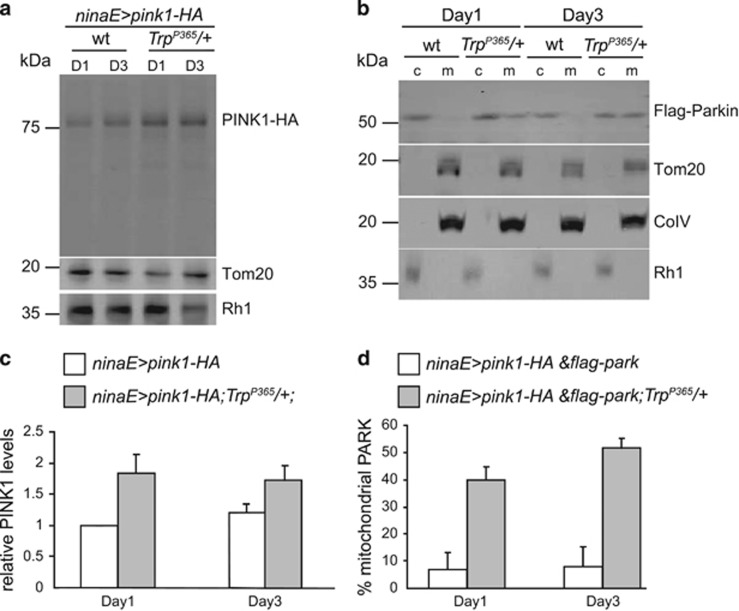
Accumulation of PINK1 and Parkin on the mitochondria of *Trp*^*P365*^ photoreceptor cells. (**a**) Western blot of head extracts from *ninaE>pink1-HA* (*ninaE-gal4/UAS-pink1-HA*) and *ninaE>pink1-HA*;*Trp*^*P365*^*/+* flies. Anti-HA antibody was used to detect PINK1-HA. D1, day 1; D3, day 3. (**b**) Western blot of fractions from fly heads of *ninaE>flag-park* (*ninaE-gal4/UAS-flag-park*) and *ninaE>flag-park*;*Trp*^*P365*^*/+* flies (day 1 and day 3). Cytosolic (**c**) and mitochondrial (**m**) fractions are indicated. Anti-Flag antibody was used to detect Flag-Parkin. (**c**) The relative level of PINK1 normalized to Tom20. (**d**) Histogram quantifying the percentage of Parkin in the mitochondria fraction. Error bars indicate S.E.M.

The PINK1/Parkin pathway is a conserved pathway that mediates mitophagy. In healthy mitochondria, PINK1 is unstable and subject to rapid degradation. In damaged mitochondria, however, PINK1 accumulates on the outer membrane of depolarized mitochondria. PINK1 then recruits cytosolic Parkin to the mitochondria and initiates the mitophagic process.^18, 39^ Thus, the PINK1 protein levels were low in wild-type photoreceptor cells that overexpressed *pink1* (*ninaE>pink1-HA*). However, the PINK1 levels were increased ~twofold in *ninaE>pink1-HA*;*Trp*^*P365*^ flies (on both day 1 and day 3) prior to the onset of massive cell death (Figures 5a and c). As PINK1 was stabilized in the *Trp*^*P365*^ mutant cells, we questioned whether stabilized PINK1 was able to recruit Parkin for the autophagic degradation of mitochondria. To test this, we assayed the percentage of Parkin bound to the mitochondria by isolating mitochondria from wild-type and *Trp*^*P365*^ flies expressing both PINK1-HA and Flag-Parkin. In the wild-type flies, most of the Parkin was localized to the cytosol, whereas ~50% of the Parkin was detected in the mitochondrial fraction of the *Trp*^*P365*^ mutant flies (Figures 5b and d). It is worth mentioning that the percentage of mitochondrial Parkin was higher in 3-day-old *Trp*^*P365*^ flies than in 1-day-old *Trp*^*P365*^ flies, indicating that the mitochondria are gradually damaged.

**Figure 6:**
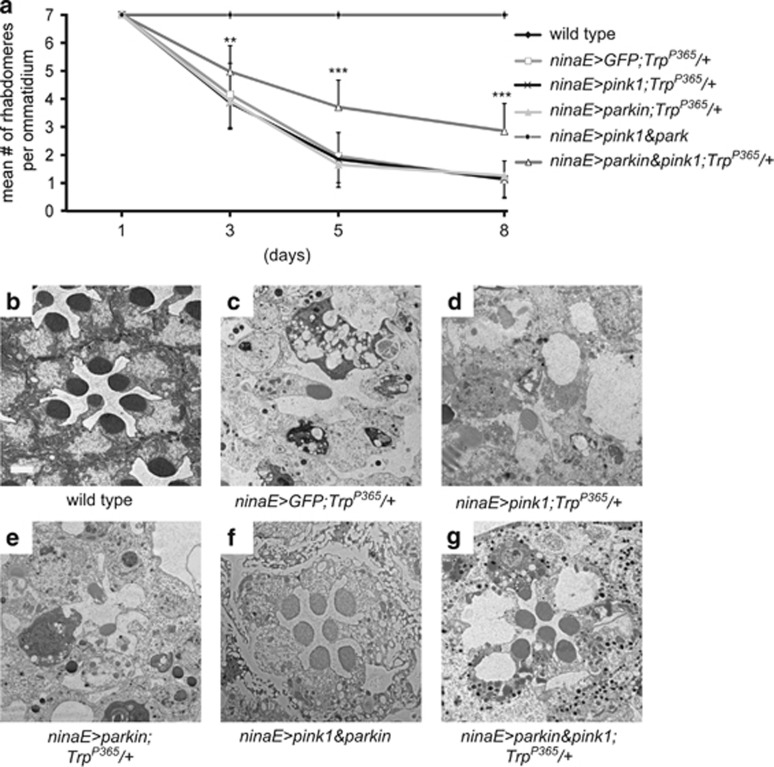
Coexpression of PINK1 and Parkin suppresses retinal degeneration in *Trp*^*P365*^ flies. (**a**) Time course of retinal degeneration was determined by optical neutralization. Quantification was based on >150 ommatidia from three eyes at each point. (**b**–**g**) Retinal morphologies of (**b**) wild-type, (**c**) *ninaE>GFP*;*Trp*^*P365*^*/+* (*ninaE-gal4/UAS-GFP*;*Trp*^*P365*^*/+*) (**d**) *ninaE>pink1*;*Trp*^*P365*^*/+* (*ninaE-gal4/UAS-pink1*;*Trp*^*P365*^*/+*), (**e**) *ninaE>park*;*Trp*^*P365*^*/+* (*ninaE-gal4/UAS-park*;*Trp*^*P365*^*/+*), (**f**) *ninaE>pink1&park*, and (**g**) *ninaE>pink1&park*;*Trp*^*P365*^*/+* flies were examined by TEM. Eight-day-old flies were used. Scale bar, 2 *μ*m. Significant differences were determined using Student's *t*-test between the *ninaE-gal4/UAS-pink1 UAS-park*;*Trp*^*P365*^*/+* and *TRP*^*P365*^ under indicated age (**P<0.01; ***P<0.001)

As the *Trp*^*P365*^ mutant cells exhibited PINK1 stabilization and the mitochondrial localization of Parkin, the evolutionarily conserved PINK1/Parkin pathway may contribute to TRP^P365^-induced cell death. To test this idea, we overexpressed PINK1 and/or Parkin in *Trp*^*P365*^ mutant photoreceptor cells via *ninaE-Gal4*. There was no obvious alleviation of the *Trp*^*P365*^-mediated degeneration of photoreceptor cells by the overexpression of PINK1 or Parkin as revealed by TEM and optical neutralization assays (Figures 6a–d and g). However, the degeneration was greatly reduced by coexpressing PINK1 and Parkin (Figures 6e–g). These results support the hypothesis that the accumulation of damaged mitochondria has a key physiological role in *Trp*^*P365*^**-**induced cell death. Further, the results suggest that the degradation of damaged mitochondria by the PINK1/Parkin-mediated mitochondria quality control pathway alleviated this type of cell death.

### The modification of retinal degeneration in the *Trp*^*P365*^ mutant by manipulation of mitochondrial fission and fusion

**Figure 7:**
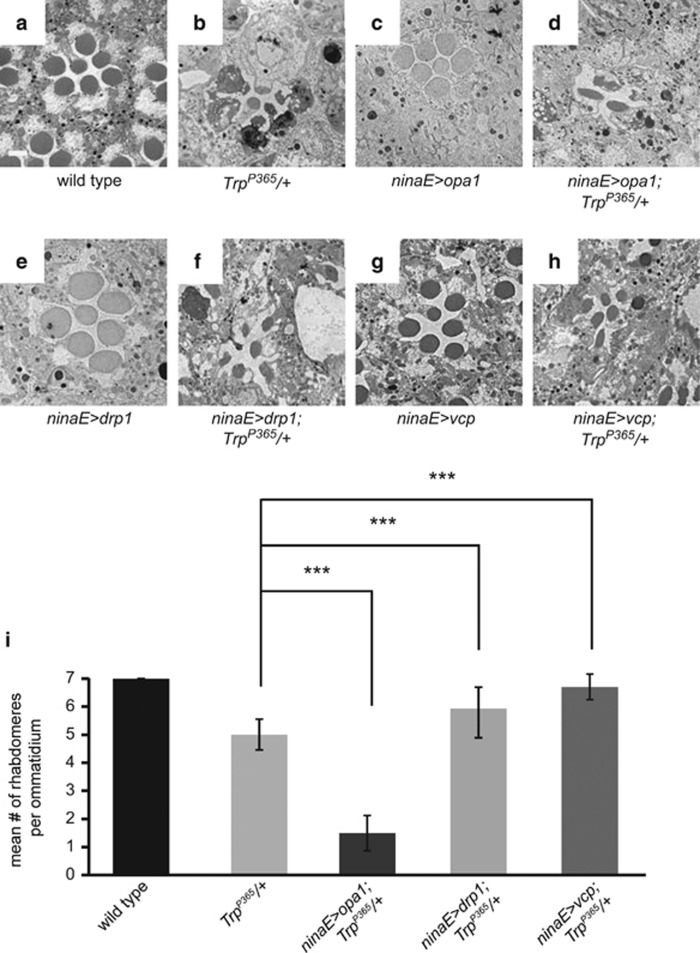
Modifying mitochondrial quality control pathways modulates the severity of photoreceptor cell death in *Trp*^*P365*^ flies. (**a**–**h**) TEM images of cross-sections from 8-day-old flies. (**a**) Wild-type, (**b**) *Trp*^*P365*^/+, (**c**) *ninaE>opa1* (*ninaE-gal4/UAS-opa1*), (**d**) *ninaE>opa1*;*Trp*^*P365*^/+, (**e**) *ninaE>drp1* (*ninaE-gal4/UAS-drp1*), (**f**) *ninaE>drp1*;*Trp*^*P365*^/+, (**g**) *ninaE>vcp* (*ninaE-gal4/UAS-vcp*), and (**h**) *ninaE>vcp*;*Trp*^*P365*^/+. Scale bar, 2 *μ*m. (**i**) Histogram of the mean number of rhabdomeres per ommatidium from 8-day-old flies. The quantification is based on the examination of TEM images of ⩾150 ommatidia from three flies. Error bars indicate S.E.M.s (***P<0.001)

Mitochondrial fission can produce small, impaired daughter mitochondria that are targeted by the mitophagic machinery. In contrast, mitochondrial fusion may dilute the impaired mitochondria and thereby prevent their mitophagic degradation.^40, 41^ Therefore, we assessed the impact of the mitochondrial fusion and fission pathways on the survival of *Trp*^*P365*^ mutant cells. This was accomplished by overexpressing Opa1 or Drp1, which are positive regulators of mitofusion and mitofission, respectively. Consistent with the PINK1 and Parkin results, the severity of retinal degeneration was alleviated by Drp1 overexpression and enhanced by Opa1 overexpression in 5-day-old *Trp*^*P365*^ flies (Figures 7a–f and i). It has recently been reported that VCP can complement PINK1 deficiency and hence it is required for the cleavage of dysfunctional mitochondria.^42, 43^ We therefore investigated whether VCP is able to protect *Trp*^*P365*^ mutant photoreceptor cells. As expected, VCP strongly protected against the death of *Trp*^*P365*^ mutant cells (Figures 7g–i). These results further support the conclusion that the mitochondrial quality control pathways have key roles in promoting cell survival in response to a cellular calcium assault.

## Discussion

The dysregulation of calcium homeostasis is involved in the pathogenesis of various neurodegenerative diseases, including rapid forms of neurodegeneration associated with ischemia or brain injury, as well as slowly progressing diseases such as Alzheimer's disease, Parkinson's disease, Huntington's disease, and amyotrophic lateral sclerosis.^9^ It is therefore of critical importance to determine the mechanisms by which calcium overload results in neuronal cell death. Mitochondrial dysfunction (e.g., the loss of mitochondrial membrane potential) has been detected in neurodegenerative diseases associated with dysregulated cellular calcium. Calcium overload is a major cause of ischemic cell death, and it has been suggested that the resulting calcium overload within the mitochondrial matrix leads to mitochondrial dysfunction.^9, 44, 45^ It has been further suggested that the mitochondrial uncoupler protects cells from glutamate-induced excitotoxicity by preventing mitochondrial calcium uptake.^46^ Moreover, the loss of MICU1, a gatekeeper of MCU-mediated mitochondrial calcium uptake, leads to constitutive calcium accumulation within mitochondria, which in turn triggers excessive ROS generation and sensitivity to cell death stress.^47^ In flies, the dominant *trp* allele, *Trp*^*P365*^, caused age-dependent retinal degeneration due to elevated levels of cellular Ca^2+^ stemming from the constitutive activation of TRP channels. TEM-based morphological analysis revealed that the mitochondria of the *Trp*^*P365*^ photoreceptor cells gradually accumulated vacuoles. These mitochondria also accumulated ROS and lost membrane potential during the degeneration process. It is important to note that removing dysfunctional mitochondrial via autophagy largely alleviated neurodegeneration in *Trp*^*P365*^ flies, thereby providing strong genetic support for the hypothesis that impaired mitochondrial function contributes to the pathogenesis of cellular calcium neurotoxicity.

As neurons are essentially incapable of generating ATP once oxidative phosphorylation is lost, the loss of mitochondrial function is fatal to neuronal cells.^6^ It has been suggested that the disruption of the mitochondrial membrane potential by mitochondrial calcium accumulation has a critical role in calcium-dependent neurotoxicity. Inhibitors of MCU prevent the loss of mitochondrial membrane potential upon the deregulation of cellular calcium homeostasis by preventing mitochondrial calcium accumulation.^48, 49^ However, owing to the high instability of such MCU inhibitors, it has not been possible to determine whether the modification of mitochondrial calcium entrance affects cellular calcium neurotoxicity. The pronounced suppression of Trp^P365^-induced neurotoxicity brought about by the introduction of the Na^+^/Ca^2+^ exchanger, CalX, provided strong evidence that the retinal degeneration in the *Trp*^*P365*^ mutant primarily resulted from cellular calcium overload. The overexpression of LETM1, a mitochondria Ca^2+^/H^+^ exchange protein, largely increased calcium uptake by the mitochondria.^30^ Using a genetic approach, we demonstrated that an increase in LETM1 function enhanced retinal degeneration in the *Trp*^*P365*^ mutant. In contrast, the expression of the ER Ca^2+^ pump, Serca, alleviated photoreceptor cell death in the *Trp*^*P365*^ mutant. This may be due to the physical and functional links between the ER calcium release channel and the mitochondrial calcium uptake systems, because increased ER calcium uptake by Serca expression reduces mitochondrial calcium accumulation.^50^ It is also possible that constitutively active TRP channels disrupt calcium homeostasis in the ER, which may lead to the activation of the ER stress pathway and hence contribute to the retinal degeneration of *Trp*^*P365*^ mutants.

Autophagy is important for the degradation of intracellularly damaged organelles and misfolded proteins.^51^ Through the degradation of protein aggregates, autophagy has a vital role in maintaining cellular homeostasis, while the upregulation of autophagy in models of neurodegenerative diseases reduces toxic protein aggregates and the associated cell death.^32, 52^ However, it is important to determine whether autophagy is capable of modulating neurodegeneration in diseases not associated with aggregated proteins. Here, we found that the genetic induction of autophagy (by inhibiting TOR or expressing Atg1) alleviated cell death associated with the *Trp*^*P365*^ mutation. Moreover, selectively inhibiting mitophagy prevented the ability of the autophagy pathways to suppress calcium-induced neurotoxicity. These data strongly support a model in which autophagy suppresses neural degeneration by removing damaged organelles and toxic protein aggregates. Therefore, therapies involving the induction of autophagy may prove generally useful in treating neurological diseases.

Mitochondria are vulnerable to toxicant exposure, while mitochondrial dysfunction is associated with the normal aging process. The timely removal of damaged mitochondria is thus critical for cellular homeostasis and function. It has been proposed that mitophagy protects against neuronal loss during the normal aging process and in neurodegeneration diseases associated with damaged mitochondria.^53, 54^ Although the alleviation of cell death by autophagy has been demonstrated in several neurodegenerative diseases, it remains unclear whether this cell-protective function is mediated by the mitophagy branch. In the *Trp*^*P365*^ retinal degeneration model, autophagy suppressed cellular calcium neurotoxicity, and this effect depended on the PINK1/Parkin pathway. Furthermore, the direct induction of mitophagy via PINK1 and Parkin overexpression also protected against Trp^P365^-induced neural degeneration. Mitochondria are highly dynamic organelles, and mitochondrial fission and fusion are capable of sorting out defective mitochondria prior to mitophagy. Thus, the modulation of mitochondrial dynamics by either increasing fission or decreasing fusion leads to the isolation and mitophagic elimination of damaged mitochondria.^55^ Consistent with PINK1/Parkin expression, increased mitofission suppressed retinal degeneration in the *Trp*^*P365*^ mutant, whereas the induction of mitofusion via the expression of Opa1 had the opposite effect. These data support a model in which PINK1/Parkin-dependent mitophagy suppresses neural neurodegeneration by removing damaged mitochondria. Our findings hence indicate that the induction of mitophagy may serve as a novel therapeutic strategy for neurodegeneration associated with mitochondrial dysfunction or cellular calcium neurotoxicity.

## Materials and Methods

### Drosophila stocks

All fly growth and crosses were performed according to standard procedures at 25 °C under a 12-h light/dark cycle. Male flies were used for all experiments. The *ninaE-atg1*, *UAS-tsc1*, and *UAS-tsc2* flies have been previously described.^33^ The *ninaE-gal4*, *UAS-GFP*, *park*^*1*^, and *UAS-drp1* flies were obtained from the Bloomington Stock Center. The *Trp*^*P365*^ and *UAS-calx* flies were obtained from Dr C Montell (University of California, Santa Barbara). The *pink1*^*B9*^, *UAS-opa1*, *UAS-pink1-HA*, and *UAS-flag-parkin* flies were from Dr J Chung (Chungnam National University School of Medicine). The *UAS-atg1* flies were obtained from Dr T Neufield (University of Minnesota). Dr X Huang (Institute of Genetics and Developmental Biology, CAS) provided the *UAS-serca* flies. The EST clones GH03311 and LP12034 were used to generate *UAS-letm1* and *UAS-vcp*, respectively. The constructs were individually injected into *w*^*1118*^ embryos, and the transformants were identified on the basis of eye color.

### Tissues for live imaging

All fly retinas were dissected in Schneider's *Drosophila* Medium (Sigma, St. Louis, MO, USA) containing 10% heat-inactivated fetal bovine serum (Invitrogen, Carlsbad, CA, USA). The mitochondrial membrane potential was measured by staining with 25 *μ*M JC-10 reagent (Enzo Life Sciences, New York, NY, USA).^56^ The fluorescence intensities at excitation/emission wavelengths of 490/525 nm and 540/590 nm were obtained with a Nikon A1 confocal microscope (Nikon, Tokyo, Japan). The mitochondrial superoxide levels were measured by staining with mitoSOX (Invitrogen).

### Transmission electron microscopy

TEM was performed on cross-sections of fly eyes as previously described.^26^ Briefly, the fly heads were dissected and pre-fixed in a PBS buffer containing 2.5% glutaraldehyde (Sigma), 4% paraformaldehyde (Sigma), and 2% tannic acid (EMS, Hatfield, PA, USA), followed by post-fixation in 1% OsO_4_. The samples were embedded in LR White Resin (EMS), and thin sections (~90 nm) were prepared at a depth of 30 *μ*m. The sections were then examined by TEM (JEM-1400; JEOL, Tokyo, Japan), and images were acquired with a Gatan camera (Model 832; Gatan, Pleasanton, CA, USA).

### Quantification of mitochondrial morphology in TEM

The identical mitochondrial morphology was measured using Image J. The longest length of a single mitochondria was measured as the length of mitochondria. The images used for the mitochondrial morphology analysis were taken with at least a × 10 000 amplification factor. After the measurement of the mitochondrial length, data were analyzed with Origin 8 frequency count. The computation dialog was from minimum 0.1 *μ*m to maximum 2 *μ*m, and the step increment was 0.2 *μ*m.

### Mitochondria isolation and western blotting

The isolation of fly mitochondria was performed as previously described,^56^ except that 20 eyes were gently homogenized in 1 ml of mitochondrial isolation buffer (250 mM sucrose, 10 mM Tris (pH 7.4), and 0.15 mM MgCl_2_). The mitochondrial (pellet) and cytosolic (supernatant) fractions were solubilized in a 50 *μ*l SDS sample buffer and then subjected to western blotting.

In order to detect HA-tagged PINK1 and Flag-tagged Parkin on western blots, the samples were homogenized in an SDS sample buffer, fractionated by SDS-PAGE, and transferred to Immobilon-FL membranes (Millipore, Billerica, MA, USA). The blots were then incubated with mouse anti-HA (Roche, Indianapolis, IN, USA) or mouse anti-Flag (Sigma) primary antibodies or control antibodies, which included mouse anti-Rh1 (Developmental Studies Hybridoma Bank, Ames, IA, USA), rabbit anti-CoIV (Abcam, Cambridge, MA, USA), and rat-anti-TOM20. The blots were subsequently incubated with IRDye 800-labeled anti-mouse IgG and IRDye 680-labeled anti-rabbit or rat IgG (LI-COR Biosciences, Lincoln, NE, USA). The signals were then detected using an Odyssey Infrared Imaging System (LI-COR Biosciences).

### Statistical analysis

All statistical analyses were performed using MS Excel and Origin 8. Unpaired two-tailed Student's *t*-tests were used.

## Abbreviations

ATP: adenosine triphosphate
ROS: reactive oxygen species
PINK1: PTEN-induced putative kinase 1
TRP: transient receptor potential
LETM1: Leucine zipper-EF-hand containing transmembrane protein 1
SERCA: sarco/endoplasmic reticulum Ca^2+^-ATPase
TORC1: target of rapamycin complex 1
TSC: tuberous sclerosis complex
Rheb: Ras homolog enriched in brain
Drp1: dynamin-related protein1
Opa1: Optic atrophy 1
VCP: valosin-containing protein
MICU: mitochondrial calcium uptake 1
MCU: mitochondrial calcium uniporter
Clax: Na^+^/Ca^2+^-exchange protein
ER: endoplasmic reticulum
atg1: autophagy related 1
UAS: upstream activator sequence
*ninaE*: *neither inactivation nor after potential E*

## References

[bib1] Berridge MJ. Neuronal calcium signaling. Neuron 1998; 21: 13–26. 9697848 10.1016/s0896-6273(00)80510-3

[bib2] Berridge MJ, Bootman MD, Roderick HL. Calcium signalling: dynamics, homeostasis and remodelling. Nat Rev Mol Cell Biol 2003; 4: 517–529. 12838335 10.1038/nrm1155

[bib3] Orrenius S, Zhivotovsky B, Nicotera P. Regulation of cell death: the calcium-apoptosis link. Nat Rev Mol Cell Biol 2003; 4: 552–565. 12838338 10.1038/nrm1150

[bib4] Starkov AA, Chinopoulos C, Fiskum G. Mitochondrial calcium and oxidative stress as mediators of ischemic brain injury. Cell Calcium 2004; 36: 257–264. 15261481 10.1016/j.ceca.2004.02.012

[bib5] Mattson MP. Excitation BolsTORs motor neurons in ALS mice. Neuron 2013; 80: 1–3. 24094096 10.1016/j.neuron.2013.09.017

[bib6] Herrero-Mendez A, Almeida A, Fernandez E, Maestre C, Moncada S, Bolanos JP. The bioenergetic and antioxidant status of neurons is controlled by continuous degradation of a key glycolytic enzyme by APC/C-Cdh1. Nature Cell Biol 2009; 11: 747–752. 19448625 10.1038/ncb1881

[bib7] Nunnari J, Suomalainen A. Mitochondria: in sickness and in health. Cell 2012; 148: 1145–1159. 22424226 10.1016/j.cell.2012.02.035 PMC5381524

[bib8] Youle RJ, van der Bliek AM. Mitochondrial fission, fusion, and stress. Science 2012; 337: 1062–1065. 22936770 10.1126/science.1219855 PMC4762028

[bib9] Rizzuto R, De Stefani D, Raffaello A, Mammucari C. Mitochondria as sensors and regulators of calcium signalling. Nat Rev Mol Cell Biol 2012; 13: 566–578. 22850819 10.1038/nrm3412

[bib10] Vergun O, Keelan J, Khodorov BI, Duchen MR. Glutamate-induced mitochondrial depolarisation and perturbation of calcium homeostasis in cultured rat hippocampal neurones. J Physiol 1999; 519(Pt 2): 451–466. 10457062 10.1111/j.1469-7793.1999.0451m.x PMC2269520

[bib11] Rapizzi E, Pinton P, Szabadkai G, Wieckowski MR, Vandecasteele G, Baird G et al. Recombinant expression of the voltage-dependent anion channel enhances the transfer of Ca^2+^ microdomains to mitochondria. J Cell Biol 2002; 159: 613–624. 12438411 10.1083/jcb.200205091 PMC2173108

[bib12] Giacomello M, Drago I, Pizzo P, Pozzan T. Mitochondrial Ca^2+^ as a key regulator of cell life and death. Cell Death Differ 2007; 14: 1267–1274. 17431419 10.1038/sj.cdd.4402147

[bib13] Rugarli EI, Langer T. Mitochondrial quality control: a matter of life and death for neurons. EMBO J 2012; 31: 1336–1349. 22354038 10.1038/emboj.2012.38 PMC3321185

[bib14] Youle RJ, Narendra DP. Mechanisms of mitophagy. Nat Rev Mol Cell Biol 2011; 12: 9–14. 21179058 10.1038/nrm3028 PMC4780047

[bib15] Greene JC, Whitworth AJ, Kuo I, Andrews LA, Feany MB, Pallanck LJ. Mitochondrial pathology and apoptotic muscle degeneration in Drosophila parkin mutants. Proc Natl Acad Sci USA 2003; 100: 4078–4083. 12642658 10.1073/pnas.0737556100 PMC153051

[bib16] Clark IE, Dodson MW, Jiang C, Cao JH, Huh JR, Seol JH et al. Drosophila pink1 is required for mitochondrial function and interacts genetically with parkin. Nature 2006; 441: 1162–1166. 16672981 10.1038/nature04779

[bib17] Park J, Lee SB, Lee S, Kim Y, Song S, Kim S et al. Mitochondrial dysfunction in Drosophila PINK1 mutants is complemented by parkin. Nature 2006; 441: 1157–1161. 16672980 10.1038/nature04788

[bib18] Narendra D, Tanaka A, Suen DF, Youle RJ. Parkin is recruited selectively to impaired mitochondria and promotes their autophagy. J Cell Biol 2008; 183: 795–803. 19029340 10.1083/jcb.200809125 PMC2592826

[bib19] Matsuda N, Tanaka K. Uncovering the roles of PINK1 and Parkin in mitophagy. Autophagy 2010; 6: 952–954. 20724841 10.4161/auto.6.7.13039 PMC3039741

[bib20] Narendra DP, Jin SM, Tanaka A, Suen DF, Gautier CA, Shen J et al. PINK1 is selectively stabilized on impaired mitochondria to activate Parkin. PLoS Biol 2010; 8: e1000298. 20126261 10.1371/journal.pbio.1000298 PMC2811155

[bib21] Vives-Bauza C, Zhou C, Huang Y, Cui M, de Vries RL, Kim J et al. PINK1-dependent recruitment of Parkin to mitochondria in mitophagy. Proc Natl Acad Sci USA 2010; 107: 378–383. 19966284 10.1073/pnas.0911187107 PMC2806779

[bib22] Wang T, Montell C. Phototransduction and retinal degeneration in Drosophila. Pflugers Arch 2007; 454: 821–847. 17487503 10.1007/s00424-007-0251-1

[bib23] Lessing D, Bonini NM. Maintaining the brain: insight into human neurodegeneration from Drosophila melanogaster mutants. Nat Rev Genet 2009; 10: 359–370. 19434080 10.1038/nrg2563 PMC2820605

[bib24] Jaiswal M, Sandoval H, Zhang K, Bayat V, Bellen HJ. Probing mechanisms that underlie human neurodegenerative diseases in Drosophila. Annu Rev Genet 2012; 46: 371–396. 22974305 10.1146/annurev-genet-110711-155456 PMC3663445

[bib25] Xiong B, Bellen HJ. Rhodopsin homeostasis and retinal degeneration: lessons from the fly. Trends Neurosci 2013; 36: 652–660. 24012059 10.1016/j.tins.2013.08.003 PMC3955215

[bib26] Wang T, Xu H, Oberwinkler J, Gu Y, Hardie RC, Montell C. Light activation, adaptation, and cell survival functions of the Na^+^/Ca^2+^ exchanger CalX. Neuron 2005; 45: 367–378. 15694324 10.1016/j.neuron.2004.12.046

[bib27] Liu K, Li Y, Liu L. Modeling calcium-overload mediated necrosis in Drosophila. Methods Mol Biol 2013; 1004: 203–213. 23733579 10.1007/978-1-62703-383-1_15

[bib28] Montell C, Rubin GM. Molecular characterization of the Drosophila trp locus: a putative integral membrane protein required for phototransduction. Neuron 1989; 2: 1313–1323. 2516726 10.1016/0896-6273(89)90069-x

[bib29] Yoon J, Ben-Ami HC, Hong YS, Park S, Strong LL, Bowman J et al. Novel mechanism of massive photoreceptor degeneration caused by mutations in the trp gene of Drosophila. J Neurosci 2000; 20: 649–659. 10632594 10.1523/JNEUROSCI.20-02-00649.2000 PMC6772429

[bib30] Jiang D, Zhao L, Clapham DE. Genome-wide RNAi screen identifies Letm1 as a mitochondrial Ca^2+^/H^+^ antiporter. Science 2009; 326: 144–147. 19797662 10.1126/science.1175145 PMC4067766

[bib31] Fleming A, Noda T, Yoshimori T, Rubinsztein DC. Chemical modulators of autophagy as biological probes and potential therapeutics. Nat Chem Biol 2011; 7: 9–17. 21164513 10.1038/nchembio.500

[bib32] Menzies FM, Fleming A, Rubinsztein DC. Compromised autophagy and neurodegenerative diseases. Nat Rev Neurosci 2015; 16: 345–357. 25991442 10.1038/nrn3961

[bib33] Wang T, Lao U, Edgar BA. TOR-mediated autophagy regulates cell death in Drosophila neurodegenerative disease. J Cell Biol 2009; 186: 703–711. 19720874 10.1083/jcb.200904090 PMC2742187

[bib34] Kim J, Guan KL. Amino acid signaling in TOR activation. Annu Rev Biochem 2011; 80: 1001–1032. 21548787 10.1146/annurev-biochem-062209-094414

[bib35] Reiling JH, Sabatini DM. Stress and mTORture signaling. Oncogene 2006; 25: 6373–6383. 17041623 10.1038/sj.onc.1209889

[bib36] D'Gama AM, Geng Y, Couto JA, Martin B, Boyle EA, LaCoursiere CM et al. mTOR pathway mutations cause hemimegalencephaly and focal cortical dysplasia. Ann Neurol 2015; 77: 720–725. 25599672 10.1002/ana.24357 PMC4471336

[bib37] Jones R. The roles of PINK1 and Parkin in Parkinson's disease. PLoS Biol 2010; 8: e1000299. 20126262 10.1371/journal.pbio.1000299 PMC2811156

[bib38] Liu S, Lu B. Reduction of protein translation and activation of autophagy protect against PINK1 pathogenesis in Drosophila melanogaster. PLoS Genet 2010; 6: e1001237. 21151574 10.1371/journal.pgen.1001237 PMC3000346

[bib39] Jin SM, Lazarou M, Wang C, Kane LA, Narendra DP, Youle RJ. Mitochondrial membrane potential regulates PINK1 import and proteolytic destabilization by PARL. J Cell Biol 2010; 191: 933–942. 21115803 10.1083/jcb.201008084 PMC2995166

[bib40] Deng H, Dodson MW, Huang H, Guo M. The Parkinson's disease genes *pink1* and *parkin* promote mitochondrial fission and/or inhibit fusion in Drosophila. Proc Natl Acad Sci USA 2008; 105: 14503–14508. 18799731 10.1073/pnas.0803998105 PMC2567186

[bib41] Poole AC, Thomas RE, Andrews LA, McBride HM, Whitworth AJ, Pallanck LJ. The PINK1/Parkin pathway regulates mitochondrial morphology. Proc Natl Acad Sci USA 2008; 105: 1638–1643. 18230723 10.1073/pnas.0709336105 PMC2234197

[bib42] Kim NC, Tresse E, Kolaitis RM, Molliex A, Thomas RE, Alami NH et al. VCP is essential for mitochondrial quality control by PINK1/Parkin and this function is impaired by VCP mutations. Neuron 2013; 78: 65–80. 23498974 10.1016/j.neuron.2013.02.029 PMC3683300

[bib43] Kimura Y, Fukushi J, Hori S, Matsuda N, Okatsu K, Kakiyama Y et al. Different dynamic movements of wild-type and pathogenic VCPs and their cofactors to damaged mitochondria in a Parkin-mediated mitochondrial quality control system. Genes Cells 2013; 18: 1131–1143. 24215292 10.1111/gtc.12103

[bib44] Szydlowska K, Tymianski M. Calcium, ischemia and excitotoxicity. Cell Calcium 2010; 47: 122–129. 20167368 10.1016/j.ceca.2010.01.003

[bib45] Cali T, Ottolini D, Brini M. Mitochondrial Ca^2+^ and neurodegeneration. Cell Calcium 2012; 52: 73–85. 22608276 10.1016/j.ceca.2012.04.015 PMC3396847

[bib46] Stout AK, Raphael HM, Kanterewicz BI, Klann E, Reynolds IJ. Glutamate-induced neuron death requires mitochondrial calcium uptake. Nat Neurosci 1998; 1: 366–373. 10196525 10.1038/1577

[bib47] Mallilankaraman K, Doonan P, Cardenas C, Chandramoorthy HC, Muller M, Miller R et al. MICU1 is an essential gatekeeper for MCU-mediated mitochondrial Ca^2+^ uptake that regulates cell survival. Cell 2012; 151: 630–644. 23101630 10.1016/j.cell.2012.10.011 PMC3486697

[bib48] Duan Y, Gross RA, Sheu SS. Ca^2+^-dependent generation of mitochondrial reactive oxygen species serves as a signal for poly(ADP-ribose) polymerase-1 activation during glutamate excitotoxicity. J Physiol 2007; 585: 741–758. 17947304 10.1113/jphysiol.2007.145409 PMC2375529

[bib49] Abramov AY, Duchen MR. Mechanisms underlying the loss of mitochondrial membrane potential in glutamate excitotoxicity. Biochim Biophys Acta 2008; 1777: 953–964. 18471431 10.1016/j.bbabio.2008.04.017

[bib50] Szabadkai G, Bianchi K, Varnai P, De Stefani D, Wieckowski MR, Cavagna D et al. Chaperone-mediated coupling of endoplasmic reticulum and mitochondrial Ca2+ channels. J Cell Biol 2006; 175: 901–911. 17178908 10.1083/jcb.200608073 PMC2064700

[bib51] Mizushima N, Levine B, Cuervo AM, Klionsky DJ. Autophagy fights disease through cellular self-digestion. Nature 2008; 451: 1069–1075. 18305538 10.1038/nature06639 PMC2670399

[bib52] Klionsky DJ, Abdalla FC, Abeliovich H, Abraham RT, Acevedo-Arozena A, Adeli K et al. Guidelines for the use and interpretation of assays for monitoring autophagy. Autophagy 2012; 8: 445–544. 22966490 10.4161/auto.19496 PMC3404883

[bib53] Palikaras K, Tavernarakis N. Mitophagy in neurodegeneration and aging. Front Genet 2012; 3: 297. 23267366 10.3389/fgene.2012.00297 PMC3525948

[bib54] Schiavi A, Maglioni S, Palikaras K, Shaik A, Strappazzon F, Brinkmann V et al. Iron-starvation-induced mitophagy mediates lifespan extension upon mitochondrial stress in *C. elegans*. Curr Biol 2015; 25: 1810–1822. 26144971 10.1016/j.cub.2015.05.059

[bib55] Twig G, Elorza A, Molina AJ, Mohamed H, Wikstrom JD, Walzer G et al. Fission and selective fusion govern mitochondrial segregation and elimination by autophagy. EMBO J 2008; 27: 433–446. 18200046 10.1038/sj.emboj.7601963 PMC2234339

[bib56] Wu K, Liu J, Zhuang N, Wang T. UCP4A protects against mitochondrial dysfunction and degeneration in pink1/parkin models of Parkinson's disease. FASEB J 2014; 28: 5111–5121. 25145627 10.1096/fj.14-255802

