# Supplementary figures and images for: PINK1 and Parkin cooperatively protect neurons against constitutively active TRP channel-induced retinal degeneration in Drosophila
Source: Cell Death Dis. 2016 Apr 7;7(4):e2179–. doi: 10.1038/cddis.2016.82 (PMC4855661; doi:10.1038/cddis.2016.82)

Figure S1

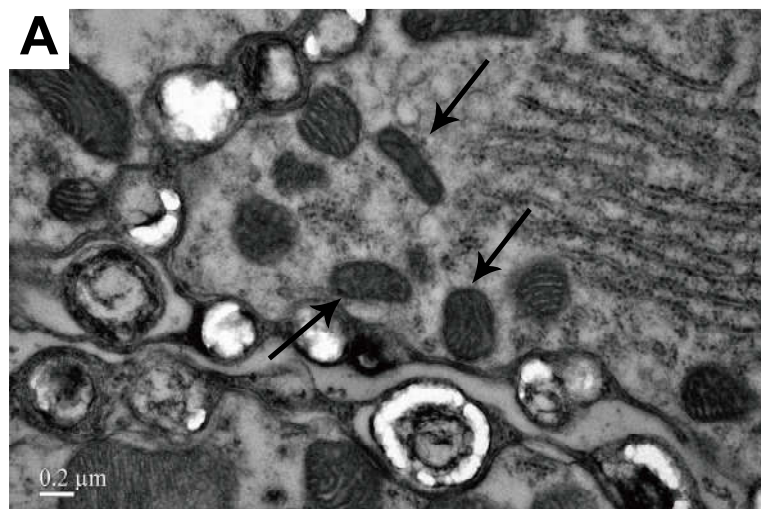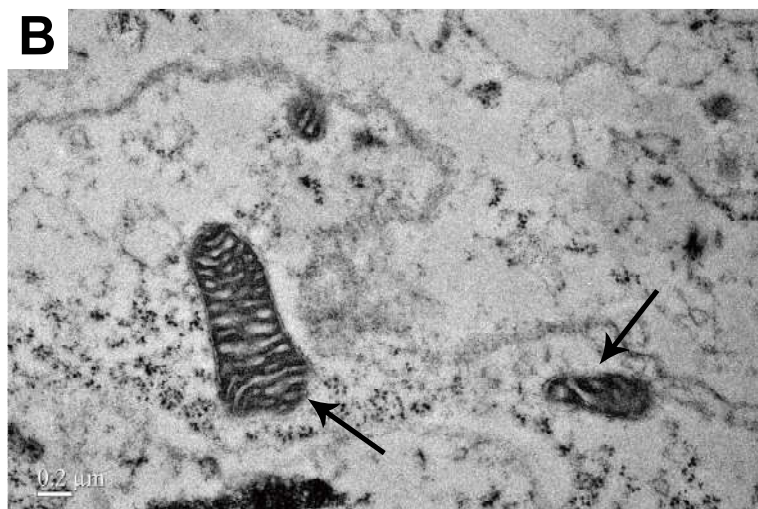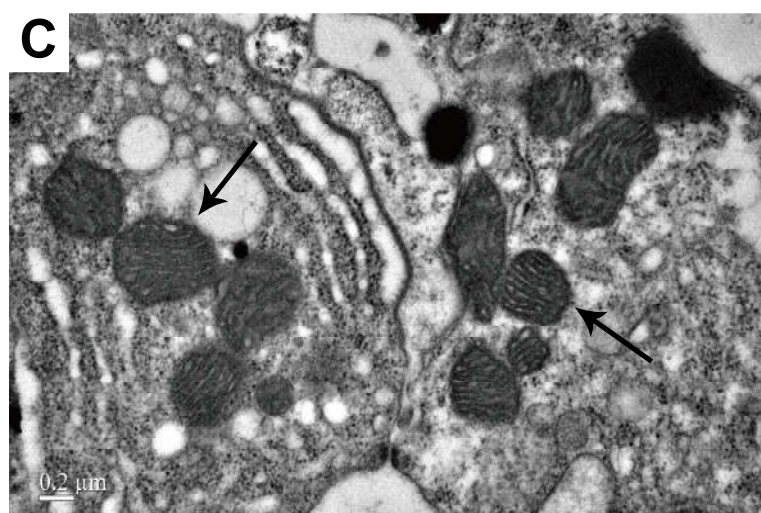

Supplement: Supplementary Figure S1 [file cddis201682x1.pdf]
