# Supplementary material for: PINK1 and Parkin cooperatively protect neurons against constitutively active TRP channel-induced retinal degeneration in Drosophila
Source: Cell Death Dis. 2016 Apr 7;7(4):e2179–. doi: 10.1038/cddis.2016.82 (PMC4855661; doi:10.1038/cddis.2016.82)
Supplement: Supplementary Figure Legend [file cddis201682x2.docx]

**Figure S1. The mitochondrial morphology of the *Trp^P365^* mutant was rescued by Atg1 expression.** The morphology of the photoreceptor cell mitochondria was examined for five-day-old (A) wild-type, (B) *Trp^P365^*/+, and (C) *ninaE-atg1;Trp^P365^*/+ flies. Arrows indicate mitochondria. Scale bar, 0.2 µm.
